# Experimental observation of the effect of immunotherapy on CD4+ T cells and Th1/Th2 cytokines in mice with allergic rhinitis

**DOI:** 10.1038/s41598-023-32507-6

**Published:** 2023-03-31

**Authors:** Yu Zhu, Juan Yu, XinHua Zhu, JiaSheng Yuan, MeiNa Dai, YouWei Bao, YinLi Jiang

**Affiliations:** Department of Otolaryngology Head and Neck Surgery, The Second Affiliated Hospital of Nanchang University, Nanchang, 330006 China

**Keywords:** Diseases, Respiratory tract diseases

## Abstract

The present study aims to investigate the effect of immunotherapy in a mouse model of allergic rhinitis (AR) and to explore the possible molecular mechanisms of action. An animal model of AR was established by sensitization and challenge of BALB/c mice with house dust mite (HDM) extract. The mice were injected subcutaneously with HDM for immunotherapy. AR nasal symptoms were evaluated according to the frequencies of nose rubbing and sneezing and the degree of rhinorrhea. The nasal mucosa and lung tissue architecture and inflammatory status by histological analysis; the infiltration of eosinophils in nasal lavage fluid (NALF) of mice was observed by Diff-Quik stain; ELISA-based quantification of serum HDM-specific IgE and TH1/TH2 cytokine concentration; and flow cytometry detected the number of serum CD4+/CD8+ cells to evaluate the mechanism of immunotherapy. It was found that after immunotherapy, the AR symptom score was reduced, the number of eosinophils in NALF was reduced, and the infiltration of inflammatory cells and tissue damage in the nasal mucosa and lung tissue were alleviated. Immunotherapy can increase the number of CD4+ T cells in the peripheral blood, increase the ratio of CD4+/CD8+ cells, increase the expression of Th1 cytokines such as IL-2 and IFN-γ, reduce the expression of Th2 cytokines such as IL-4 and IL-5. The results showed that repeated intraperitoneal injection of crude extract of HDM for sensitization, followed by nasal drops can effectively construct a mouse model of AR, and subcutaneous injection of immunotherapy in mice can reduce allergic inflammation in model mice and improve the inflammatory infiltration of the nasal cavity in allergic rhinitis. Immunotherapy can reduce the expression of inflammatory factors in AR, improve Th1/Th2 balance, and may play a role in the treatment of AR by improving the function of immune cells.

## Introduction

Allergic rhinitis (AR) is a chronic inflammatory disease of the nasal mucosa mediated by IgE after individual exposure to allergens^1,2^. The key effector cells of AR inflammation response are eosinophils, which are derived from bone marrow pluripotent hematopoietic stem cells and can migrate from bone marrow to peripheral blood and local nasal mucosal tissues and play an important role in the occurrence and development of AR^3^. In addition, T helper (Th)2 polarization makes an important contribution to the pathogenesis of allergic diseases. AR CD4+ T cells are insensitive to apoptotic stimuli, destroying their apoptotic machinery. The fact may prolong the lifespan of Th2 cells, contribute to the development of Th2 polarization, and promote the development of AR^4^.

Epidemiological studies have shown that the incidence of AR is increasing globally, especially in developed countries, and currently affects 10–40% of adults and 2–25% of children worldwide^5^. Thus effective treatment is particularly important. AR treatment is built on three cornerstones: allergen avoidance (challenging to achieve), drugs (mostly antihistamines and intranasal corticosteroids), and allergen-specific immunotherapy (AIT). Among them, AIT is the first-line treatment for AR, which aims at the etiology and can change the natural course of the disease through the immunomodulatory mechanism^6^. The two most commonly prescribed routes for AIT are subcutaneous immunotherapy (SCIT) and sublingual immunotherapy (SLIT)^7^. One of the mechanisms of AIT altering allergen-specific T cells is the switch from a Th2 to a Th1 cell-dominated immune response (immune deviation). Many clinical trial results in the past have proved this viewpoint—the immune effect of immunotherapy can reduce the secretion of Th2 cytokines and increase the secretion of Th1 cytokines^8,9^. Although there have been many reports on the immunomodulatory mechanism of AIT on AR, its mechanism of action is still not completely clear, and further research is needed.

Animal models of AR are an important means to study AR. At present, most models are induced by Ovalbumin (OVA), but it is merely an experimental preclinical model using a purified protein that lacks the properties of natural allergens^10^. Natural allergens such as House dust mites (HDMs) very frequently lead to AR as well as HDMs allergy is the most common worldwide. HDM allergy accounts for about 50% of sensitizations that occurred in AR patients^6^. Subcutaneous AIT with HDM mite allergens is significantly effective in patients with allergic rhinitis and asthma who are allergic to dust mites^11^. The HDM-sensitized mouse model is more similar to human AR, so the establishment of the HDM extract-induced AIT mouse model is very important in the research of AR and immunotherapy. At present, there are many reports on the establishment of asthma mouse models by HDM at home and abroad, but few on the establishment of AR mouse models. In this study, HDM extract was used to sensitize and challenge BALB/c mice, and HDM extract was used for subcutaneous immunotherapy^12^, in an attempt to construct a specific immunotherapy mouse model induced by HDM extract. To preliminarily prove the effect of AIT on immune function. Then further study how it affects immune function and look for further intermediate mechanisms.

Based on this, this study aimed to construct mouse models of AR and AIT induced by HDM, and explore the effect of immunotherapy on the AR through the HDM-induced AIT mouse model. The possible mechanisms of immunotherapy in AR mice were explored by observing nasal symptoms, histomorphology, HDM-sIgE concentration in peripheral blood, changes in CD4+ T cells, and Th1/Th2 cytokines.

## Results

### Immunotherapy affected nasal symptoms in the AR mouse model

**Figure 1:**
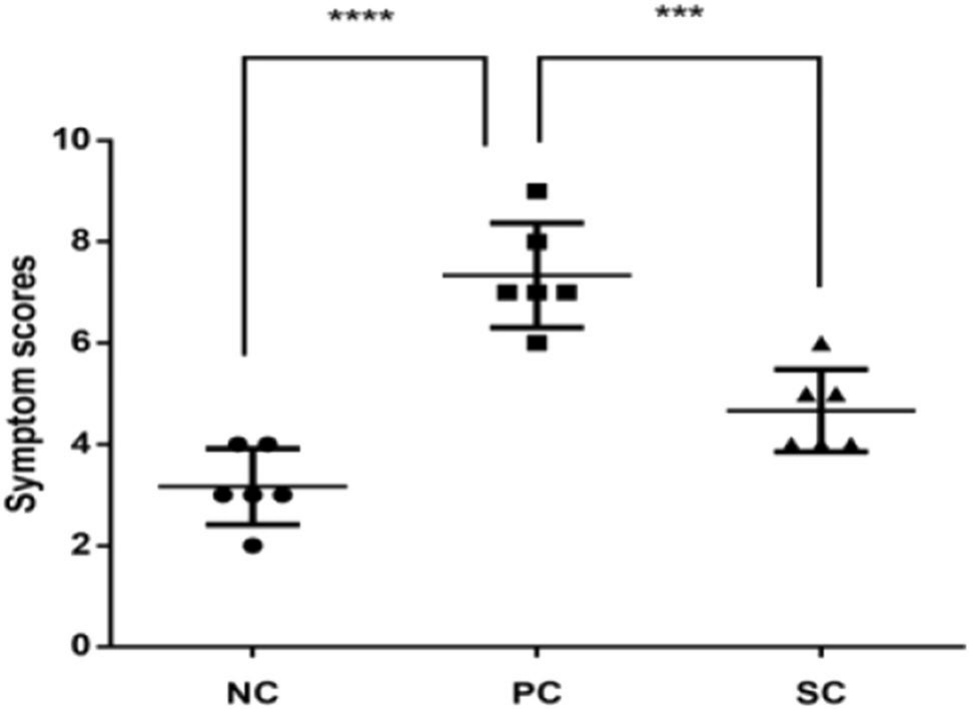
Statistical results of nasal symptom scores in different groups. Data are expressed as mean ± standard deviation. Compared with the NC group, SC group, and PC group, ***P < 0.001, ****P < 0.0001.

To examine the effect of immunotherapy on nasal symptoms, we scored the AR symptoms according to the frequency of sneezing events and nose rubbing and the degree of rhinorrhea in mice 30 min after the last nasal stimulation(maximum total score = 9, Table 2). Compared with the NC group (Table 1), the PC group had a total nasal symptom score of ≥ 5, which was considered to reflect AR. However, AR symptoms in SC mice were significantly improved compared with PC mice (Fig. 1). These results suggest that HDM extract could induce the symptoms of AR in PC mice, and immunotherapy can effectively improve the symptoms of AR in mice.

**Table 1:** Experimental mouse grouping.

Experimental mouse grouping	Groups
Normal control groups	NC
AR mouse models	PC
Immunotherapeutic mouse models	SC

### Immunotherapy affected serum HDM-sIgE in AR mice

**Figure 2:**
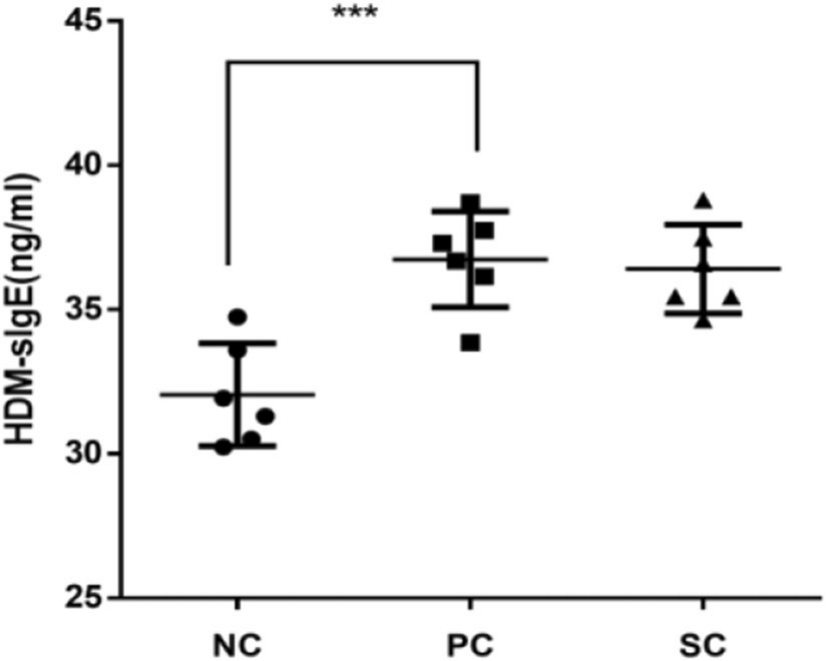
The concentration of HDM-specific IgE in serum was determined by ELISA. ***P < 0.001.

As shown in Fig. 2, compared with NC mice, the serum HDM-sIgE level of PC mice was significantly increased, and the difference was statistically significant. Yet there was no significant decrease in serum HDM-sIgE in SC mice compared with PC mice. The results showed that HDM-sIgE increased in HDM-sensitized AR mice, but did not decrease after immunotherapy.

### Immunotherapy affected the number of inflammatory cells in NALF of AR mice

**Figure 3:**
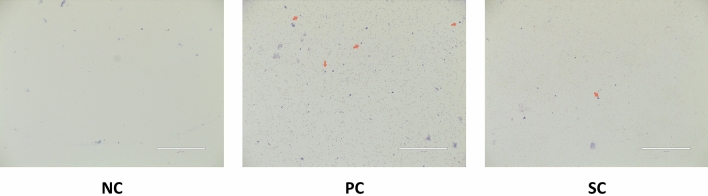
Diff-Quik was used to detect the infiltration of inflammatory cells in nasal lavage fluid (the orange arrows indicate eosinophils).

The number of eosinophils in NALF of the PC group was significantly higher than that of the NC group, while it was significantly decreased in the SC group (Fig. 3), suggesting that immunotherapy could improve the inflammatory infiltration in the nasal cavity of AR patients, and thus improve the AR symptoms.

### Effect of immunotherapy on the nasal mucosa and lung histology of AR mice

**Figure 4:**
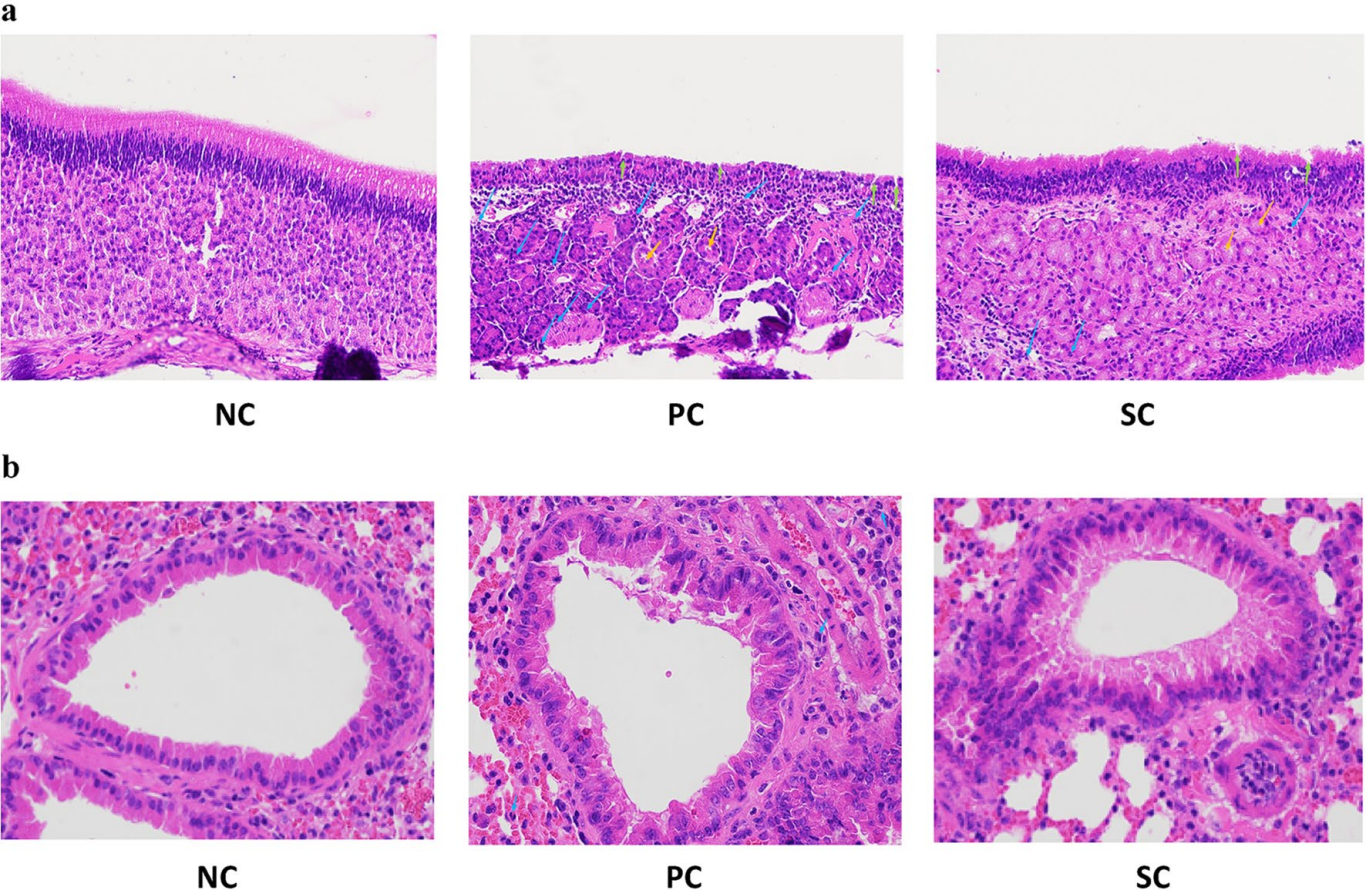
Effects of immunotherapy on histology of nasal mucosa (**a** 200×) and lung tissue (**b** 400×) in AR mice. The pathological changes of the nasal mucosa and lung tissue were observed by HE staining (the green arrows indicate epithelial damage, the orange arrows indicate glands, the blue arrows indicate eosinophils).

To explore whether HDM modeling affects the morphology and structure of the main organs of mice and the effect of immunotherapy on the histology of nasal mucosa and lung of AR mice, the nasal mucosa and lung samples were collected after the modeling was completed, and the pathological morphological changes of each organ were observed and compared by HE staining. It was found that the nasal mucosa of NC mice was intact and clear, without obvious infiltration of inflammatory cells and glandular hyperplasia. PC and SC group mice had glandular hyperplasia, epithelial damage, and detachment, submucosal swelling, and infiltration of inflammatory cells. However, the damage degree of the nasal mucosal structure of mice in the SC group was significantly less than that in the PC group (Fig. 4a). The results showed that AR mice had severe nasal mucosa inflammation, and HDM immunotherapy could reduce the damage degree of nasal mucosa in AR mice, and reduce the proliferation of glands and infiltration of inflammatory cells.

Similar to the situation of the nasal mucosa, compared with the NC group, there were a large number of inflammatory cells and eosinophil infiltration in the lung tissues of mice in the PC group, while the infiltration of inflammatory cells in the lung tissues of mice in the SC group was significantly reduced (Fig. 4b). This result further proves the theory of "one airway, one disease", and AR is the local manifestation of the systemic allergic inflammatory response, and HDM immunotherapy can reduce allergic inflammatory reaction in the lung.

### Effect of immunotherapy on Th1/Th2 cytokines in the serum of AR mice

**Figure 5:**
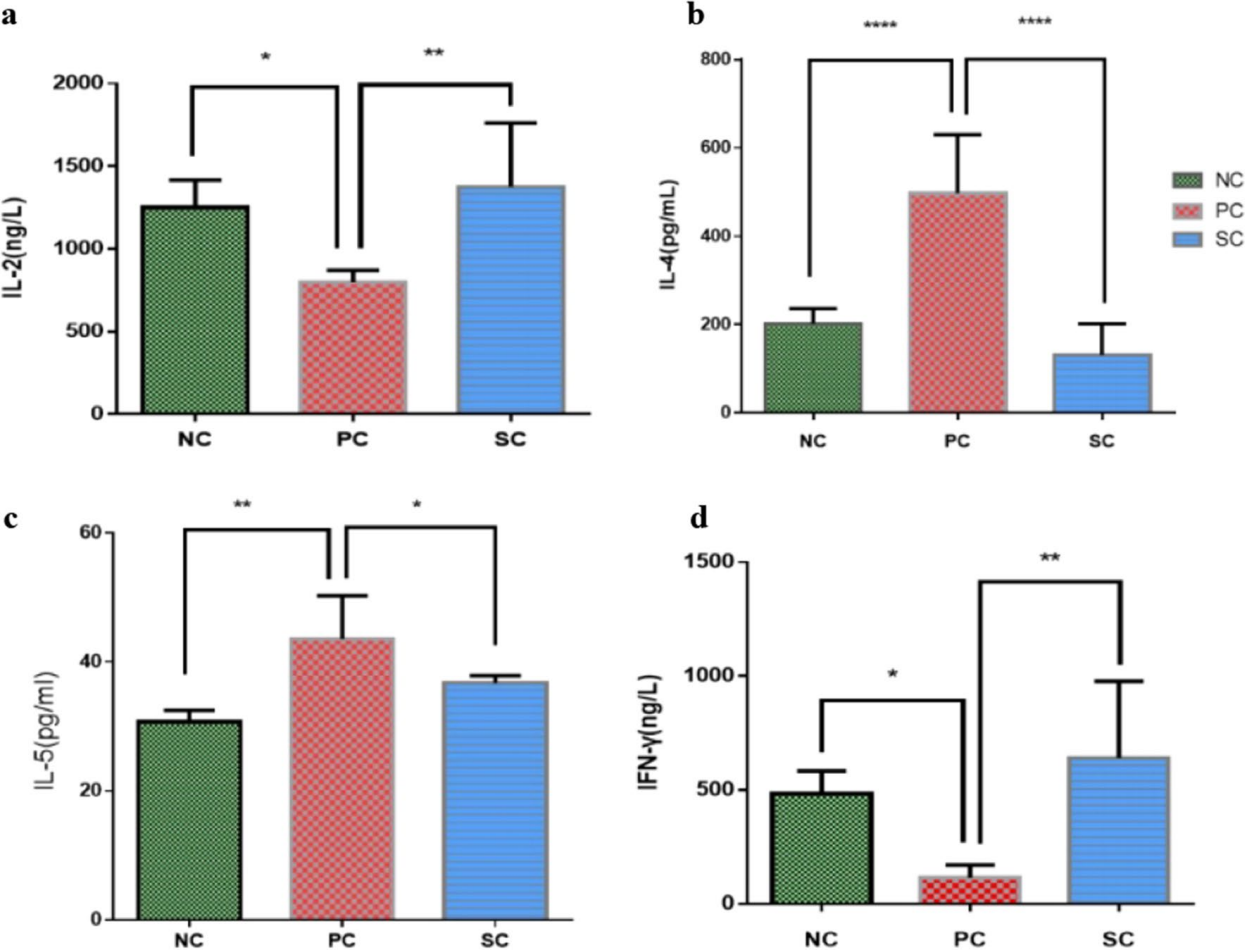
The content of cytokines in the serum of each group was determined by ELISA. Figure (**a**), (**b**), (**c**), and (**d**) show the levels of IL-2, IL-4, IL-5, and IFN-γ, respectively. *P < 0.05, **P < 0.01, ****P < 0.0001.

Compared with the control group, serum IL-4, and IL-5 were significantly increased in the PC group, and the levels of IL-2 and IFN-γ were significantly decreased. As well as, compared with the PC group, IL-4, and IL-5 in the SC group decreased significantly, while IL-2 and IFN-γ increased (Fig. 5). The above results further verified that AR was an allergic disease mainly involving Th2 cells, and that after immunotherapy, AR could improve the Th1/Th2 cell balance and thus exert effects in the treatment of AR.

### Immunotherapy affected the number of serum CD4+ T cells and the ratio of CD4+/CD8+ cells in AR mice

**Figure 6:**
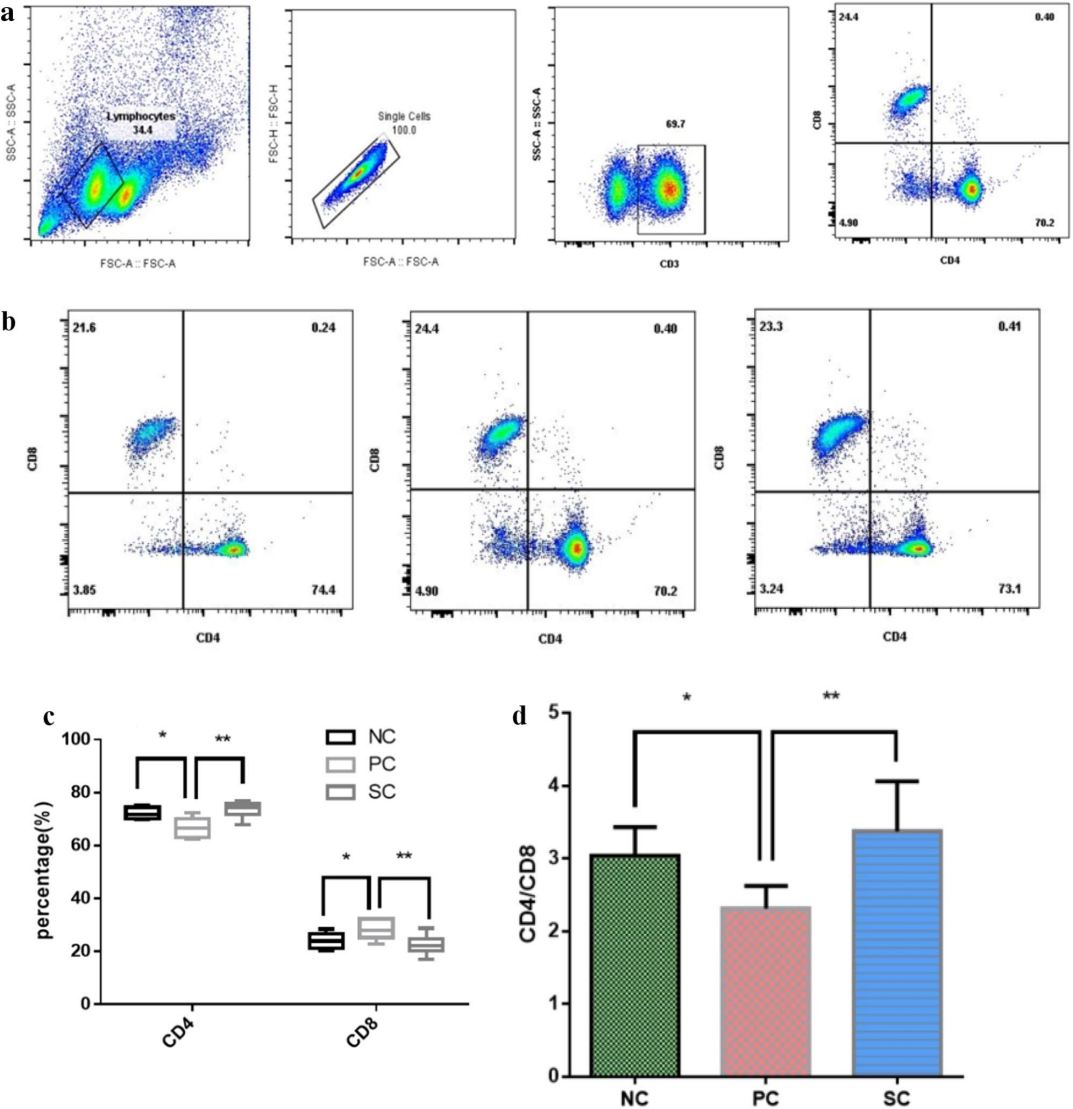
Immunotherapy can affect the number of CD4+ T cells and the ratio of CD4+/CD8+ cells in the peripheral blood of AR mice. Flow cytometry was used to determine the number of CD4+ and CD8+ T cells in the peripheral blood of mice in each group (**b**). The circle-gate strategy is shown in Figure (**a**). (**c**) is the percentage of CD4+ T and CD8+ T cells in peripheral blood, and d is the ratio of CD4+ to CD8+ cells in each group. *P < 0.05, **P < 0.01.

Flow cytometry was used to detect the number of CD4+ T cells and CD8+ T cells in the peripheral blood of mice in each group. Compared with the NC group, the number of CD4+ T cells decreased significantly and the number of CD8+ T cells increased significantly. Compared with the PC group, the number of CD4+ T cells increased significantly and the number of CD8+ T cells decreased significantly in the SC group after immunotherapy (Fig. 6a–c). The ratio of CD4+/CD8+ cells was decreased in the PC group compared with the NC group, whereas the ratio of CD4+/CD8+ cells was significantly increased in the SC group compared with the PC group (Fig. 6d). The results indicated that immunotherapy could increase the number of CD4+ T cells in peripheral blood, decrease the number of CD8+ T cells, and increase the ratio of CD4+/CD8+ cells. It was suggested that immunotherapy might treat AR by improving the function of immune cells.

## Discussion

AR is a global health problem that causes major illnesses and disabilities worldwide. Currently, it affects up to 40% of the population worldwide and its prevalence is still rising rapidly^13^. AIT is the first-line treatment for AR, which aims at the etiology and can change the natural course of the disease through the immunomodulatory mechanism. It is still very limited to study of the pathogenesis of AR and immunotherapy using simple specimens such as peripheral blood and nasal secretions of patients. Therefore, animal models play an important role in the study of AR. The successful establishment of the AR animal model plays an important role in the study of the etiology, pathology, diagnosis, and treatment of AR in humans. The successful establishment of allergen the specific immunotherapy mouse model is very important to study the therapeutic effect and mechanism of immunotherapy. Studies have shown that the use of BALB/c mice to construct an allergic airway inflammation model can induce a good Th2-biased immunological response. And female BALB/c mice are more sensitive to allergens than male BALB/c mice and are more prone to allergic symptoms^14,15^. Most of the currently used AR animal models are constructed using OVA, but OVA is not an allergen of allergic airway inflammation, and OVA induces immune tolerance without the use of immune adjuvants. Therefore, the use of inhaled allergens in the environment such as dust mites, pollen, cockroaches, fungi, etc. to construct animal models is more beneficial for the study of allergic airways^10^. Hence, in this study, female BALB/c mice were selected to establish an AR mouse model with HDM as an allergen. We used the HDM extract via intraperitoneal injection followed by an intranasal challenge. Compared with the control group, AR symptoms appeared in the experimental group, and the inflammatory cells in HDM-sIgE and NALF in the peripheral blood increased, which confirmed each other with the inflammatory manifestations such as epithelial cell shedding, inflammatory cell increase, and gland hyperplasia in the pathology of the nasal mucosa and lung tissue. It is consistent with the pathological characteristics of human AR and can be further used to evaluate the effect of specific immunotherapy. In this experiment, after subcutaneous immunotherapy with HDM extract, the times of scratching and sneezing in mice were reduced, and the histomorphology was reduced. It was preliminarily verified that immunotherapy could improve the inflammatory infiltration of the AR nasal cavity, thus improving the symptoms of AR. And the successful establishment of an allergen-specific immunotherapy mouse model was preliminarily proved. However, HDM-sIgE in peripheral blood did not significantly decrease after immunotherapy compared with mice in the experimental group. Can we find other allergen-specific markers to prove the effectiveness of immunotherapy?

Efficient host defense against invading pathogenic microorganisms is achieved through the coordination of complex signaling networks that link the innate and adaptive immune systems. Upon interaction with cognate antigen presented by antigen-presenting cells, the naive CD4 + T (NT) cells can differentiate into T effector subsets, and the classical ones are Th1 cells and Th2 cells. Th1 cells are characterized by their production of IFN-γand IL-2 and are involved in cellular immunity; Th2 cells produce IL-4, IL-5, and IL-13 and are required for humoral immunity. The balance between Th1 and Th2 cells plays a vital role in allergic and autoimmune disorders by mediating the secretion of Th1-related cytokines and Th2-related cytokines^16,17^. At present, it is unanimously agreed that the Th cell immune imbalance theory is the main pathogenesis of AR, and AR is an allergic inflammation mainly caused by Th2 immune response due to the imbalance of Thl/Th2 immunity caused by the action of inhaled allergens on the body^18,19^. The results of this experiment showed that the levels of Th2 cytokines such as IL-4 and IL-5 in the peripheral blood of rats in the PC group were increased, while the levels of Th1 cytokines IL-2 and IFN-γ were decreased, which was consistent with the pathogenesis of AR (Th1/Th2 imbalance theory)^17^. The imbalance of Th1/Th2 cells in the SC group was improved, and immune tolerance was induced, which was consistent with the clinical research of immunotherapy^20^. The differentiation of CD4+ T cells into Th2 cells causes an imbalance of Th1/Th2, which leads to AR. After immunotherapy, the differentiation of CD4+ T cells into Th2 cells is inhibited, and the Th1/Th2 cell balance is improved. However, the mechanism is still not clear.

T lymphocytes play an important role in the immune response. T lymphocytes can be divided into CD4+ and CD8+ according to their surface differentiation antigens. CD4+ is a helper T cell (TH), which can preferentially differentiate and trigger phagocyte-mediated host defense response, and has the function of helper T and B lymphocytes response. It plays an extremely important role in the fight against intracellular pathogen infection; CD8+ is an inhibitory T cell (TS), which is an inhibitory cell that inhibits the production of antibodies by B cells. When the number of TH cells is decreased and/or that of TS cells is increased, the ratio of the two cells will be changed, resulting in the decreased immune function of the body. The CD4+/CD8+ ratio can directly reflect the disorder of host T cell subsets and indirectly understand the cellular immune function of the body. Allergic diseases are immunologic disorders, such as AR disease caused by a decreased CD4/ CD8 ratio and enhanced CD 8+ T cell activation. Immunotherapy acts by modifying CD4+ T-cell responses either by immune deviation, T-cell anergy and/or both^21–23^. Lee et al.^24^ showed that the CD4/CD8 ratio in the peripheral blood during acute asthma attacks was significantly higher than that of controls, with a significant reduction after treatment. This showed that improving the body’s immune function was of great significance for the treatment of acute bronchial asthma. Some scholars may speculate that CD8+ T cells induce early cytotoxicity and edematous inflammation, while CD4+ T cells act later by inducing more modulatory effects^25^. Is it the same in AR? In this study, AR had decreased CD4+ T cell expression, increased CD8+ expression, and decreased CD4+/CD8+ cell ratio. After immunotherapy, the number of CD4+ T cells in peripheral blood increased, the number of CD8+ T cells decreased, and the ratio of CD4+/CD8+ cells increased. We established a mouse model of AIT induced by HDM extract that was similar in immunological and clinical parameters to human AR and related symptoms after treatment with AIT^26^. This is essential for the search for more effective AIT protocols and the generation of new and more effective AR treatments.

Whether immunotherapy may regulate the immune function of the body by regulating Th cell subsets, and ultimately reshape the immune response into immune tolerance. Due to the short time of immunotherapy in this study, the depth of the study is not enough, and it is only limited to the preliminary discussion. Further studies on how immunotherapy regulates the mechanism of action of Bregs and Tregs need to be conducted at the cellular level and gene levels.

## Materials and methods

### Mice

6 to 8 weeks old female SPF BALB/c mice were obtained from the Changsha Tianqin Biotechnology Co., LTD. The breeding environment was constant temperature and humidity, 12/12 h daylight circulation, and sterile feed and drinking water were provided. All procedures were approved by the Welfare Ethics Committee of Nanchang University. All experiments were performed by relevant named guidelines, regulations, and the ARRIVE guidelines.

### Mouse AR model

**Figure 7:**
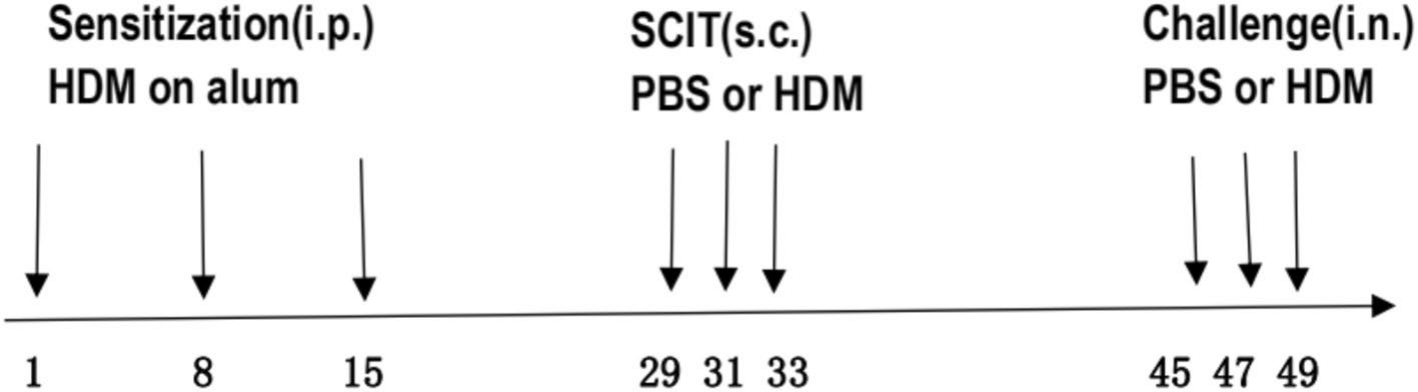
Schematic representation of the experimental allergic rhinitis model and treatment protocol.

Mice were randomly divided into immunotherapy (SC), AR (PC), and normal control groups (NC) (n = 6 per group). The AR and immunotherapeutic mouse models were established by sensitization and challenge with HDM extract^10^ (Der P1:183.25 mcg/vial, Dry Weight: 17.4 mg, Protein: 4.53 mg) (Greer Labs, USA). The mice were sensitized using an intraperitoneal injection of 100 μl PBS containing 5 μg HDM allergen extract and 20 μl aluminum hydroxide on days 1, 8, and 15. The mice in the SC and PC groups are challenged on days 45, 47, and 49 by intranasal administration of 25 μg HDM allergen extract dissolved in PBS (total volume of 20 μl), while the NC groups received PBS. 24 h after the final administration of day 49, all mice were euthanized and routine samples were taken. As shown in Fig. 7.

### Subcutaneous immunotherapy treatment

The effect of SCIT was assessed by subcutaneous treatment with HDM extract after sensitization and before the challenge. The SCIT reagent was injected subcutaneously through the cervical back. 250 μg HDM extract per 100 μl, balance in PBS. The reagents need to be prepared on the spot. The mice of the SC group are SCIT treated on days 29, 31, and 33, by subcutaneous injections of the SCIT reagent. Meanwhile, the PC and NC groups use the same dosages of PBS per mouse injection.

### Nasal symptoms changes

The nasal symptom score was based on three features, specific scoring criteria are as follows: (i) sneezing: 0, none; 1, 1–3 sneezes per 30 min; 2, 4–10 sneezes per 20 min; 3, ≥ 11 sneezes per 20 min; (ii) nasal mucus: 0, none; 1, mucus in nostrils; 2, mucus outflow from nostrils; 3, mucus outflow to face; and (iii) nasal itching: 0, none; 1, nose rubbing 1–2 times per min; 2, nose rubbing 3–5 times per min; 3, nose rubbing > 6 times per min. The three scores were added to give a total score between 0 and 9 points. Animals recording a total score of ≥ 5 points were considered to have AR (Table 2). The observation time for symptom scoring is 30 min from the moment of the last challenge.

**Table 2:** Nasal symptom scoring in the AR mouse model.

Score	Nasal symptoms		
	Number of sneezes per 30 min	Nasal mucus	Number of nasal rubbing per minute
0	None	None	None
1	1–3	Nostril	1–2
2	4–10	Outflow nostril	3–5
3	≥ 11	Flow to face	≥ 6

### Peripheral blood collection and HDM-sIgE determination

After successful modeling, the mice were anesthetized with isoflurane, their whiskers and eyelashes were trimmed, their heads and faces were fixed and the surface skin was tightened, so that the eyeballs on the blood collection side were exposed and prominent. The eyeballs were removed with ophthalmic tweezers, and peripheral blood was collected with a centrifuge tube. Blood samples were allowed to stand for 2 h at room temperature and then centrifuged at 3200 rpm for 15 min at 4 °C to obtain serum. HDM-sIgE in the serum was measured using an Enzyme-linked immunosorbent assay (ELISA) kit (Wuhan Shenko experimental technology co., ltd, China), according to the manufacturer’s instructions.

### Nasal lavage fluid (NALF) was collected and eosinophil infiltration was observed

The mice were decapitated and the mandibles were cut off along the lateral side of the bilateral oral fissure, exposing the nasopharynx. Precooled PBS was transferred from the nasopharynx of mice to the anterior nasal cavity through the posterior nostril, and an EP tube was used to catch the outflow irrigation fluid at the nostril, and repeatedly rinsed twice. The supernatant was discarded after centrifugation at 4 °C for 15 min at 5000 rpm, and the cell precipitate was suspended by 200 µl PBS. NALF smear, after natural drying treatment, immersed in methanol and fixed for the 20 s. Diff-Quik I (Solarbio life sciences, China) staining solution was used for 5 to 10 s and Diff-Quik II staining solution for 10–20 s. Immediately after washing, the infiltration of eosinophils was observed under a microscope while wet.

### Histological study

For histological analysis, insert the ophthalmic scissors vertically and gently into the nasal cavities on both sides, cut the nasal back, lift the nasal bone upward, expose the nasal septum and nasal cavity of mice, take the nasal septum mucosa and fix it in 10% neutral formalin, and then place the mice in 75% alcohol to fully infiltrate and disinfect, fix the mice with a syringe needle and foam plate, extract the lung, fully strip the surrounding fat, blood vessels, bronchus, and connective tissue, and fix it in the fixative solution. Fixed nasal and lung tissues were embedded in paraffin and tissue sections, 4 mm thick, were fixed to microscope slides and deparaffinized. It was stained with hematoxylin–eosin (H&E) and then analyzed under light microscopy (HITACHI, Japan). Nasal and lung histopathological changes were analyzed.

### ELISA

Serum was collected the same as the way described in the HDM-sIgE section. Levels of IL-2, IL-4, IL-5, and IFN-γ were measured using ELISA kits (Wuhan Shenko experimental technology co., ltd, China) according to the manufacturer’s instructions.

### Flow cytometry analysis

The number of CD4+ T cells and the ratio of CD4+/CD8+ cells in peripheral blood detected by flow cytometry: anticoagulated whole blood of mice was taken and added with 1 µl of antibodies ANTI-MO CD3E 145-2C11 FITC, ANTI-MO CD4 GK1.5 PE, ANTI-MO CD8A 53–6.7 APC (eBioscience, UK). Incubate at room temperature away from light for 30 min, add 300 µl erythrocyte lysate, put on ice for 10 min, and mix gently twice in between. After the red blood cells are lysed, the solution should be clear and transparent. Centrifuge at 450*g* 4 °C for 5 min, carefully absorb and discard the supernatant to collect cells; Add 200 µl of red blood cell lysate, and vortex gently to resuspend the white blood cells. The cells were precipitated by centrifugation at 450*g* for 5 min at 4 °C, and the supernatant was carefully absorbed and discarded, joining PBS, 500 µl, 2000 r/min, 3 min, twice. Finally, 300 µl FBS was added, and the cells were re-suspended. Before being put on the machine, it was passed through a 200-mesh filter screen and transferred to a flow tube for testing (Beckman, USA).

### Statistical analysis

All data in this experiment were statistically analyzed by GraphPad Prism 7.0, and the measurement data in the experimental data were expressed as (mean ± SEM). All data were analyzed by normality test and variance homogeneity analysis. One-way analysis of variance was used if the data conformed to the normal distribution and the requirement of variance homogeneity. If not, the P value was calculated by a nonparametric multiple rank sum test. Values of P < 0.05 were considered statistically significant.

## Supplementary Information

Supplementary Tables.

## Data Availability

All data generated or analyzed during this study are included in this article (and its Supplementary Information files).
