# Author Correction: Experimental observation of the effect of immunotherapy on CD4 + T cells and Th1/Th2 cytokines in mice with allergic rhinitis

**DOI:** 10.1038/s41598-023-35538-1

**Published:** 2023-05-22

**Authors:** Yu Zhu, Juan Yu, XinHua Zhu, JiaSheng Yuan, MeiNa Dai, YouWei Bao, YinLi Jiang

**Affiliations:** Department of Otolaryngology Head and Neck Surgery, The Second Affiliated Hospital of Nanchang University, Nanchang, 330006 China

Correction to: *Scientific Reports* 10.1038/s41598-023-32507-6, published online 31 March 2023

The original version of this Article contained an error in the Acknowledgements section.

“This work was supported by the National Natural Science Foundation of China (Grant No. 8206040058); and the Natural Science Foundation of Jiangxi Province, China (Grant No. 20202BABL206064).”

now reads:

“This work was supported by the National Natural Science Foundation of China (Grant No. 82060186); and the Natural Science Foundation of Jiangxi Province, China (Grant No. 20202BABL206064).”

The original article has been corrected.

