# Supplementary material for: Experimental observation of the effect of immunotherapy on CD4+ T cells and Th1/Th2 cytokines in mice with allergic rhinitis
Source: Sci Rep. 2023 Mar 31;13:5273. doi: 10.1038/s41598-023-32507-6 (PMC10066377; doi:10.1038/s41598-023-32507-6)
Supplement: Supplementary file 1 — Supplementary Tables. [file 41598_2023_32507_MOESM1_ESM.doc]

**Supplementary** **table 1**

Symptom score of mice after the last challenge（`x±s）

| group | score |
| --- | --- |
| NC | 3.167±0.756 |
| PC | 7.333±1.033^****^ |
| SC | 4.667±0.816^***^ |

Compared with the NC group, SC group，and PC group, ***P < 0.001, ****P < 0.0001

| group | HDM-sIgE |
| --- | --- |
| NC | 32.048±1.782 |
| PC | 36.738±1.661^***^ |
| SC | 36.403±1.538 |

**Supplementary** **table 2**

Concentration of HDM-sIgE in peripheral blood（`x±s）

Compared with the NC group, PC group, ***P<0.001

**Supplementary** **table 3**

Concentrations of IL-2, IL-4, IL-5 and IFN-γ in peripheral blood（`x±s）

| group | IL-2 | IL-4 | IL-5 | IFN-γ |
| --- | --- | --- | --- | --- |
| NC | 1090.909±116.865 | 182.300±17.504 | 33.030±1.442 | 422.462±55.818 |
| PC | 799.818±85.497^*^ | 488.633±46.013^****^ | 40.500±3.065^**^ | 136.252±8.465^*^ |
| SC | 1155.343±152.288^**^ | 168.75±40.364^****^ | 36.208±0.902^*^ | 556.297±46.888^**^ |

The PC group compared to the NC group, the SC group compared to the PC group, *P＜0.05，**P＜0.01，****P＜0.0001

**Supplementary** **table 4**

CD4+T cells, CD8+T cells and the ratio of CD4+/CD8+ cells in peripheral blood of mice（`x±s）

| group | CD4+T | CD8+T | CD4+/CD8+ |
| --- | --- | --- | --- |
| NC | 72.183±2.473 | 24.050±2.979 | 3.040±0.390 |
| PC | 66.817±3.816^*^ | 28.183±3.688^*^ | 2.624±0.314^*^ |
| SC | 73.650±3.072^**^ | 22.500±3.774^**^ | 4.061±0.691^**^ |

Compared with the NC group, the SC group and the PC group, *P < 0.05, **P < 0.01
